# Editorial: The role of immunophenotype in tumor immunotherapy response

**DOI:** 10.3389/fgene.2023.1222064

**Published:** 2023-06-15

**Authors:** Yujuan Qin, Jian Song

**Affiliations:** School of Basic Medicine, Youjiang Medical University for Nationalities, Baise, China

**Keywords:** immunotherapy, tumor microenvironment, bioinformatics, diagnosis, prognosis

The tumor microenvironment (TME) plays an important role in tumor malignant progression, immune escape, and treatment resistance (Huang et al., 2022). It is composed of components such as stromal cells, immune cells and their secreted factors, vascular endothelial cells, and extracellular matrix. The composition of these components in the TME is the basis for determining the invasion and metastasis ability of tumors, and the function of immune cells in the TME is closely related to the clinical prognosis of tumor patients (Giraldo et al., 2019). Studies have shown that immune response in the TME is a key factor involved in multiple stages of disease progression and thus has a major impact on the future development of clinical oncology interventions (Chen et al., 2015). To explore novel therapeutic options related to the tumor immune microenvironment, scholars in the recent studies have studied some molecular markers of tumor immune checkpoints (Hu et al., 2022), prognosis, and treatment (Hu et al., 2021; Cai et al., 2023), which are presented in the current Research Topic.

Sun et al. used bioinformatics to reveal the relationship between oxidative stress-related lncRNAs and lung adenocarcinoma (LUAD). They used LASSO regression and COX proportional hazard model to further identify 16 oxidative stress-related lncRNAs and establish a risk model. The overall survival (OS) was longer in the low-risk group than in the high-risk group of LUAD. Additionally, the abundance of plasma B cells in the high-risk group was higher, revealing the potential of targeting B cells as tumor immunotherapy.

Xu et al. used various databases to investigate the relationship between LMO3 and prostate cancer (PCa). They discovered that the expression of LMO3 in PCa was decreasing compared to that in normal prostate tissue. The lower the expression of LMO3, the worse the prognosis of PCa. Furthermore, enrichment analysis (GSEA) revealed that LMO3 was involved in extracellular matrix and immune response in PCa. The assessment of LMO3 expression and T-cell checkpoint confirmed that LMO3 played a crucial role in immune evasion of PCa.

Huang et al. used the tumor mutation burden (TMB) score to distinguish between “cold tumors” and “hot tumors” in clear cell renal cell carcinoma (ccRCC) through RNA sequencing data. They found significant differences between high-risk and low-risk ccRCC groups and between tumor subtypes. Additionally, the high-risk group and the low-risk group of ccRCC showed different sensitivities to first-line drugs. The TME of the high-risk group enriches more Tregs and CD8^+^ cells to aid in the immune escape of the tumor.

Li et al. used traditional Chinese medicine and tumor databases to analyze the therapeutic effect of curcumin on melanoma (SKCM) and the correlation between core gene enrichment and various metabolic processes. The results of the cell scratch test showed that the degree of inhibition of SK-MEL-1 at different time periods was different, indicating a potential anti-migration effect. Curcumin was found to promote apoptosis in the TUNEL assay. Traditional Chinese medicine network pharmacology has demonstrated that curcumin can be used as a molecular marker for the diagnosis and prognosis of SKCM.

Jiang et al. explored the necroptosis-related lncRNAs (NLRs) in bladder cancer (BLCA) and used LASSO to screen out the relevant NLRs and establish a risk model. The results showed that the survival time of low-risk NLRs was significantly longer than that of high-risk NLRs. The IC_50_ drug sensitivity of the two groups in MIBC was also evaluated, and the high-risk group was found to be more sensitive to specific chemotherapy drugs. Additionally, they found that CD4^+^ T cells were the target cells that influenced the efficacy of BLCA immunotherapy, indicating that CD4^+^ T cells could predict the clinical effect of anti-PD-L1 and have a better prognosis.

The studies described previously were primarily based on a combination of bioinformatics analysis and validation using retrospective data. The overall clinical challenge remains to identify specific drivers associated with particular phenotypes in the tumor immune microenvironment and to validate them in prospective studies. However, we believe that the current study will stimulate a deeper understanding of cancer development and progression and provide new ideas for clinical prognosis and treatment of cancer.
